# Effects of liraglutide vs. lifestyle changes on soluble suppression of tumorigenesis-2 (sST2) and galectin-3 in obese subjects with prediabetes or type 2 diabetes after comparable weight loss

**DOI:** 10.1186/s12933-022-01469-w

**Published:** 2022-03-11

**Authors:** Paola Simeone, Romina Tripaldi, Annika Michelsen, Thor Ueland, Rossella Liani, Sonia Ciotti, Kåre I. Birkeland, Hanne L. Gulseth, Augusto Di Castelnuovo, Francesco Cipollone, Pål Aukrust, Agostino Consoli, Bente Halvorsen, Francesca Santilli

**Affiliations:** 1 Department of Medicine and Aging, and Center for Advanced Studies and Technology (CAST), “G. D’Annunzio” University Foundation, Via Luigi Polacchi, 66013 Chieti, Italy; 2 Research Institute of Internal Medicine, Oslo University Hospital Rikshospitalet, University of Oslo, Oslo, Norway; 3 Department of Transplantation Medicine, Institute of Clinical Medicine, University of Oslo and Oslo University Hospital, Oslo, Norway; 4 Department of Endocrinology, Morbid Obesity and Preventive Medicine, Oslo University Hospital, Oslo, Norway; 5 Department of Chronic Diseases and Ageing, Norwegian Institute of Public Health, Oslo, Norway; 6 Mediterranea Cardiocentro, Naples, Italy; 7 Section of Clinical Immunology and Infectious Diseases, Oslo University Hospital, Oslo, Norway

**Keywords:** Liraglutide, Diabetes, sST2, Gal-3, Markers, Cardiac fibrosis

## Abstract

**Background:**

Soluble suppression of tumorigenesis-2 (sST2) and galectin (Gal)-3 are two biomarkers related to inflammation, metabolic disturbances and to myocardial fibrosis that characterize several cardiac pathological conditions. Increased circulating levels of these molecules have been associated with risk of cardiovascular death. Treatment with liraglutide, a glucagon-like peptide 1 analog, is associated with weight loss, improved glycemic control, and reduced cardiovascular risk. We wanted to assess (I) potential differences between subjects with prediabetes or type 2 diabetes mellitus (T2DM) and healthy controls in sST2 and Gal-3 circulating levels, and their relationship with glycemic control and markers of beta cell function and myocardial injury; (II) whether liraglutide treatment modulates these markers in subjects with prediabetes or early T2DM independently of weight loss; (III) whether baseline levels of any of these two molecules may predict the response to liraglutide treatment.

**Methods:**

Forty metformin-treated obese subjects (BMI ≥ 30) with prediabetes [impaired fasting glucose (IFG) or impaired glucose tolerance (IGT) or both (n = 23)] or newly diagnosed T2DM (n = 17), were randomized to liraglutide or lifestyle counseling until achieving a comparable weight loss (7% of initial body weight). Thirteen subjects were enrolled as healthy controls for baseline sST2 and Gal-3 levels.

**Results:**

Baseline sST2 levels were comparable between controls and obese patients (p = 0.79) whereas Gal-3 levels were significantly higher in patients as compared to controls (p < 0.001). Liraglutide treatment, but not weight loss achieved by lifestyle counseling, decreased plasma sST2 levels (− 9%, beta = − 14.9, standard deviation 6.9, p = 0.037) while Gal-3 levels did not change. A reduction in serum hs-Troponin I was observed after intervention, due to a 19% (p = 0.29) increase in the lifestyle arm, and a 25% decrease (p = 0.033) in the liraglutide arm (between-group difference p = 0.083). Lower baseline Gal-3 levels predicted a better improvement in beta cell function after liraglutide treatment.

**Conclusions:**

Liraglutide-induced reduction in sST2 and possibly hs-TnI suggests that in obese patients with prediabetes or early T2DM this drug may have a positive effect on (cardiac) fibrosis, whereas plasma level of Gal-3 before liraglutide initiation may predict response to the drug in terms of beta cell function improvement.

*Trial registration* Eudract: 2013-001356-36

**Supplementary Information:**

The online version contains supplementary material available at 10.1186/s12933-022-01469-w.

## Background

Cardiac fibrosis is characterized by the accumulation of extracellular matrix (ECM) proteins in the cardiac interstitium and is associated with many cardiac pathophysiologic conditions. Both experimental and clinical evidence suggests that cardiac fibrotic alterations may be reversible [1].

Soluble suppression of tumorigenesis-2 (sST2) and galectin-3 (Gal-3) are two biomarkers of fibrogenesis and inflammation, as well as of their interactions, thought to reflect myocardial fibrosis [2]. Multiple cohort studies support the use of plasma levels of these molecules to track the disease state giving rise to heart failure and to obtain important prognostic information [3–5]. Additionally, these markers of ECM remodelling may also help identify subsets of patients who are most likely to benefit from various therapies [6].

The soluble form sST2, an interleukin-1 (IL-1) receptor family member, is secreted into the circulation and functions as a “decoy” receptor for IL-33, inhibiting the cardioprotective IL-33/ST2 signaling. It was first classified as an indicator of myocyte stress, but it is mainly produced in extracardiac tissues in response to inflammatory and fibrotic stimuli [7, 8]. In patients with chronic heart failure (HF) of ischemic etiology, an inverse association was shown between sST2 levels and flow-mediated dilation, reflecting endothelial function in vivo [9]. Higher level of sST2 has also been reported as an independent predictor of major adverse cardiovascular events and all-cause death in patients with coronary artery disease with and without T2DM [10] and of cardiovascular death and HF-related hospitalizations in patients with chronic HF [11], independent of ejection fraction [12].

Gal-3, a β-galactoside-binding lectin with carbohydrate-recognition domain, is another molecule with important role in ECM remodelling and inflammation and has also be related to angiogenesis, inflammation or fibrosis [13] and myocardial failure [2].

Liraglutide, an analogue of glucagon-like peptide-1 (GLP-1), is indicated for the treatment of type 2 diabetes mellitus (T2DM). A double-blind trial revealed that in diabetic patients at high cardiovascular risk, the occurrence of a composite endpoint including rate of death from cardiovascular causes, nonfatal myocardial infarction, or nonfatal stroke was lower with liraglutide than with placebo [14]. Improvement of cardiac fibrosis, as suggested by in vitro and in vivo evidence [15, 16], might be among the possible mechanisms beyond this finding. Along these lines, a randomized, placebo-controlled study reported histological improvement of non-alcoholic steatohepatitis with liraglutide [17]. However, although inflammation and fibrogenesis and their interaction are important features of T2DM, data on sST2 and in particular in diabetes are scarce.

## Methods

Aim of this work was to understand the effect of liraglutide *vs.* lifestyle changes-induced weight loss (7% of initial body weight) on markers involved in cardiovascular stress/tissue fibrosis in obese subjects with prediabetes or early T2DM. To reach this purpose we evaluated the circulating levels sST2, Gal-3 and serum high-sensitivity troponin I (hs-TnI), before and after comparable weight loss in the two treatment arms. Soluble ST2 and Gal-3 have been related to metabolic disturbances and we therefore also examined the relationship between these molecules *and* diabetes control and markers of beta cell function, and their potential role as predictors of treatment response.

This was a post hoc analysis conducted using stored serum and plasma samples from a randomized, double-blind, controlled, parallel-arm study designed to assess, in obese subjects with impaired glucose tolerance (IGT) and/or impaired fasting glucose (IFG) or early T2DM, the effects of an equal degree of weight loss, achieved by either lifestyle changes or liraglutide, on cardiometabolic variables [18]. The protocol and patient characteristics have been previously described in detail [18].

**Fig. 1:**
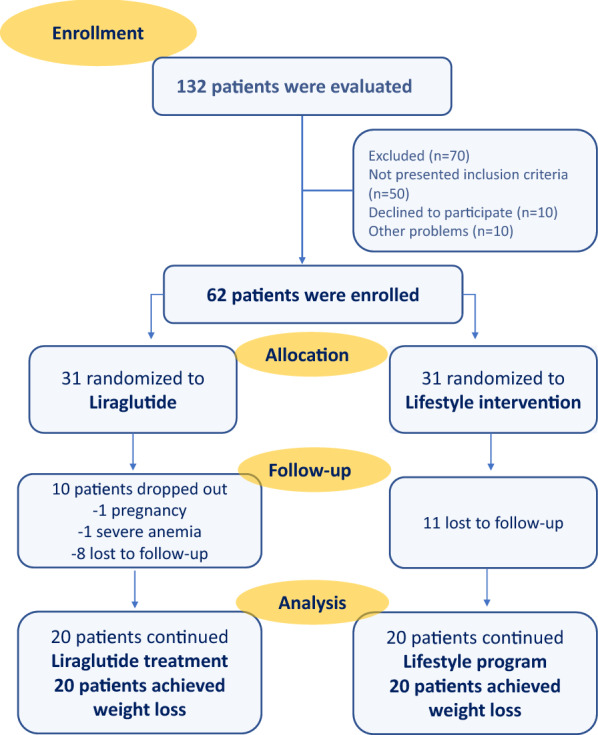
Flow chart. Flow chart of enrolment of participants in the study

Forty obese patients with prediabetes (IFG and IGT) or early T2DM [15] were enrolled at the Obesity and Diabetes Clinics of Chieti University Hospital (Fig. 1). In addition, 13 subjects, without obesity, diabetes mellitus or prediabetes and not on pharmacological treatment, were also enrolled as controls. All study visits and procedures took place at the Clinical Research Center within Department of Medicine and Aging, Center for Advanced Studies and Technology (CAST), University of Chieti, Italy. Each patient provided written informed consent to participate, and the Protocol was approved by the Ethics Committee of the University of Chieti, and the Regional Ethical Committee in South-Eastern Norway approved the import of blood for laboratory assessments in Norway.

This study was performed under the Good Clinical Practice regulations (Good Clinical Practice for Trial on Medicinal Product—CPMP/European Commission-July 1990; Decreto Ministeriale 27.4.1992—Ministero della Sanità) and the Declaration of Helsinki (Hong Kong 1989). In addition, by signing the present protocol, participants in the study committed themselves to adhere to local legal requirements.

### Outcome and study visits

After signing the informed consent, subjects were evaluated. Both visits included: clinical evaluation; abdominal MRI for adipose tissue quantification with visceral adipose tissue (VAT) and subcutaneous adipose tissue (SAT) assessment. A frequent sampling oral glucose tolerance test (OGTT) was performed for assessment of insulin sensitivity (Matsuda index, HOMA-IR), and beta cell function (OGTT beta index).

### Randomization

After a baseline evaluation, patients were randomized in a 1:1 ratio to receive liraglutide or lifestyle counselling. Study medication was supplied to the research pharmacy by Novo Nordisk as liraglutide 6.0 mg/mL in 3-mL prefilled pen injectors. Liraglutide treatment was administered by daily subcutaneous injection at bedtime and initiated with 0.6 mg per day (first week) and titrated over a 3-week period to 1.2 mg daily (second week) to 1.8 mg daily (third week), based on the clinical response and side effects. The non-attainment of the 1.8 mg dose level did not constitute a withdrawal criterion.

The computer-generated random allocation sequence was prepared by the trial statistician in blocks of four participants. Based on the order of inclusion in the study, subjects were assigned a consecutive random number, and then allocated to one of the two treatment groups.

The weight loss goal for all the participants was to lose 7% of initial body weight (calculated since the baseline visit, at the time of randomization).

### Analytical measurements

#### Biological material collection

At inclusion in the study and after the achievement of the weight loss goal, venous blood samples were collected and frozen at − 80 °C for subsequent biochemical measurements.

#### OGTT with frequent sampling

On a subset of patients, (11 randomized to lifestyle and 15 randomized to liraglutide) we evaluated circulating sST2 and Gal-3 levels before (T0), and 60, 90 and 120 min after a 75 g glucose load. The analysis was performed both at baseline and after achieving the weight loss.

#### Biochemical measurements

Plasma and serum levels of sST2 and Gal-3 were measured by enzyme-linked immunosorbent assays from R&D Systems (Stillwater, MN) according to the Manufacturer's instructions and with intra- and inter-assay coefficients of variation < 10%. Serum levels of hs-TnI were measured by ARCHITECT STAT immunoassay by Abbott, according to International Federation of Clinical Chemistry and Laboratory Medicine (IFCC) requirements for hs-TnI analytical characteristics with assay coefficients of variation < 10% (according to the Manufacturer's instructions).

### Statistical analysis

We planned a study with 20 experimental subjects and 20 control subjects. With this sample, we are able to detect a true difference in the mean response of experimental and control subjects of − 27% or + 27% of the standard deviation of a specific outcome/biomarker with probability (power) 0.9. The Type I error probability associated with this test of the null hypothesis that the population means of the experimental and control groups are equal is 0.01; this value has been established for taking into account in some way the multiple comparisons problem.

Comparisons of variables between groups (prediabetic plus diabetic patients *vs.* controls) and between arms (liraglutide *vs.* lifestyle counseling) were performed by χ^2^ tests or Mann–Whitney U tests. Spearman rank correlation test was used to assess relationships among continuous variables. The primary continuous outcomes were compared between arms by ANCOVA. The dependent variables were deltas of sST2 and Gal-3 relative to baseline value. All the variables significantly different between the two arms despite randomization were included in the analysis (sST2 basal levels, triglycerides, waist circumference, VAT).

Longitudinal changes over time of sST2 and Gal-3 in OGTT test were assessed with a general linear model on repeated measurements. Dependent variable was percent change. The effect of both treatment group and time were evaluated.

Association of baseline Gal-3 levels and improvement in beta cell function was evaluated by Spearman’s Correlation. The difference of effect (liraglutide *vs.* lifestyle) on beta cell improvement (Delta beta-index) was evaluated in two groups according to baseline Gal-3 levels (above or under the median). Two-tailed probabilities were used for testing statistical significance, and p < 0.05 was considered statistically significant. For multiple comparisons we used Bonferroni correction, for 5% statistical significance we considered p < 0.017. All calculations were carried out using SPSS (SPSS, Chicago, IL, USA).

## Results

### Baseline characteristics

The clinical and biochemical baseline characteristics of controls and patients before liraglutide or lifestyle induced weight-loss intervention are described in Table 1.

**Table 1:** Clinical baseline characteristics of controls and patients randomized to liraglutide or lifestyle induced weight-loss intervention

Variable	Controls (n = 13)	Pre-liraglutide (n = 20)	Pre-lifestyle (n = 20)	Pre-liraglutide vs. pre-lifestyle p-value	Controls vs. pre-liraglutide	Controls vs. pre-lifestyle
Age (years)	66.0 (58–69)	55 (48–63)	52 (50–57)	0.481	0.010	0.005
Gender (male), n (%)	7 (53)	11 (55)	10 (50)	1.00	1.00	1.00
BMI (kg/m^2^)	22.8 (21.5–26.6)	36.7 (34.7–40.9)	35.0 (31.3–40.3)	0.244	< 0.001	< 0.001
Weight (kg)	78.0 (61.5–86.0)	109 (95–115)	96 (86–106)	0.056	< 0.001	0.001
Type 2 diabetes, n (%)	0 (0)	10(50)	7(35)	0.523	0.002	0.027
Waist (cm)	NA	116.5 (112.0–128.5)	110.0 (100.4–119.2)	0.040	–	–
WHR	NA	0.97 (0.92–1.04)	0.9 (0.9–1)	0.321	–	–
Systolic BP (mmHg)	NA	144.5 (130–153)	134.0 (122.2–143.2)	0.144	–	–
Diastolic BP (mmHg)	NA	83.0 (78.0–87.5)	80.0 (70.0–83.7)	0.315	–	–
Hypertension, n (%)	NA	17 (85)	12 (60)	0.155	–	–
Dyslipidemia, n (%)	NA	9 (45)	10 (50)	1.00	–	–
CVD, n (%)	NA	1 (5)	5 (25)	0.182	–	–
Previous MI, or revascularization, n (%)	NA	0 (0)	1(5)	1.00	–	–
Previous TIA/stroke, or revascularization, n (%)	NA	1 (5)	1 (5)	1.00	–	–
PAD, n (%)	NA	1 (5)	0(0)	1.00	–	–
Carotid stenosis, n (%)	NA	0 (0)	4 (20)	0.106	–	–
Microvascular disease, n (%)	NA	0 (0)	0 (0)	–	–	–
Total cholesterol (mmol/L)	5.2 (4.6–6.3)	4.4 (3.6–5.0)	4.4 (3.8–4.6)	0.337	0.024	0.003
LDL cholesterol (mmol/L)	2.79 (2.56–3.15)	2.45 (1.76–3.26)	2.58 (1.99–3.00)	0.715	0.144	0.187
HDL cholesterol (mmol/L)	1.8 (1.7–2.3)	1.2 (1.0–1.4)	1.1 (1.0–1.4)	0.668	0.001	< 0.001
Triglycerides (mmol/L)	1.00 (0.60–1.38)	1.4 (0.9–2.2)	1.0 (0.8–1.3)	0.026	0.024	0.490
Amylase (U/L)	NA	56.5 (53.5–70.75)	62.5 (52.5–77.2)	0.583	–	–
Lipase (U/L)	NA	105.0 (66.2–117.5)	134.5 (66.5–173.2)	0.149	–	–
Fasting plasma glucose (mmol/L)	NA	5.2 (4.9–5.9)	5.3 (5.0–5.7)	0.989	–	–
HbA1c (%)	5.5 (5.3–5.6)	5.95 (5.62–6.70)	6.1 (5.6–6.5)	0.862	< 0.001	< 0.001
HbA1c (mmol/mol)	37 (34–38)	42 (38–50)	43 (38–48)	0.862	< 0.001	< 0.001
Fasting plasma insulin (uU/ml)	–	13.35 (9.62–20.92)	10.7 (7.5–21.7)	0.394	–	–
Creatinine (mg/dL)	0.82 (0.71–1.06)	0.70(0.63–0.81)	0.8 (0.7–0.9)	0.089	0.064	0.755
hs-C-reactive protein (mg/dL)^a^	1.15 (0.81–2.68)	0.45 (0.27–0.86)	0.3 (0.1–0.5)	0.354	–	–
AST (U/L)	27.0 (22.8–32.5)	29.0 (24.2–39)	33.0 (27.5–43.5)	0.316	0.302	0.054
ALT (U/L)	24.0 (16.3–27.8)	41.0 (36.2–46.5)	50.0 (33.2–66.5)	0.394	< 0.001	< 0.001
Metformin, n (%)	NA	20(100)	20 (100)	1.00	–	–
ACE-I, n (%)	NA	4 (20)	3 (15)	1.00	–	–
ARBs, n (%)	NA	7 (35)	6 (30)	1.00	–	–
Diuretics, n (%)	NA	7 (35)	5 (25)	0.731	–	–
B-block, n (%)	NA	7(35)	4 (20)	0.480	–	–
CCA, n (%)	NA	0 (0)	1 (5)	1.00	–	–
Statins, n (%)	NA	2 (10)	5 (25)	0.407	–	–
Fibrates, n (%)	NA	0 (0)	0 (0)	–	–	–
Omega 3, n (%)	NA	1 (5)	0 (0)	1.00	–	–
Proton pump inhibitors, n (%)	NA	3 (15)	3 (15)	1.00	–	–
ASA, n (%)	NA	1 (5)	3 (15)	0.605	–	–
SAT (cm^2^)	NA	434.1 (317.9–527.2)	374.9 (254.2–455.3)	0.311	–	–
VAT (cm^2^)	NA	324.2 (257.0–386.9)	254.5 (180.2–318.9)	0.046	–	–
sST2 (ng/mL)	11.5 (9.3–16.1)	15.01 (10.46–16.73)	10.62 (9.20–12.76)	0.008	0.161	0.347
Gal-3 (ng/mL)	1.69 (1.35–2.27)	2.97 (1.88–3.98)	3.15 (2.66–4.43)	0.461	0.004	< 0.001

*BMI* body mass index, *BP *blood pressure, *IGT* impaired glucose tolerance, *IFG* impaired fasting glucose, *WHR* waist-hip ratio, *CVD* cardiovascular disease, *MI* myocardial infarction, *TIA* transient ischemic attack, *PAD* peripheral artery disease, *ACE-I* ACE-inhibitors, *ARBs* angiotensin receptor blockers, *B-block* beta-blockers, *CCA* calcium channel antagonists, *ASA* acetylsalicylic acid, *SAT* subcutaneous-adipose-tissue, *VAT* visceral-adipose-tissue, *hs-CRP* high sensitivity-CRP

Data are median (25th–75th percentile)

^a^Creactive protein referred to high sensitivity-CRP (hs-CRP) only in patients

**Fig. 2:**
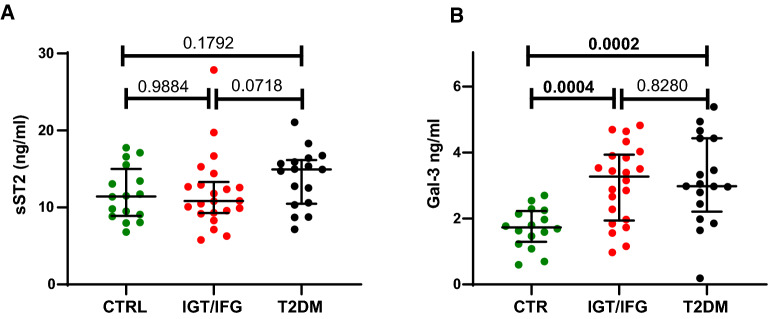
Baseline plasma sST2 and Gal-3 levels. Comparison of baseline levels of sST2 (**A**) and Gal-3 (**B**) between controls and patients randomized in the study

Baseline sST2 levels were comparable between patients and controls randomized in the study (p = 0.786) whereas Gal-3 levels were significantly higher in patients as compared to controls (p < 0.001) (Fig. 2). Similarly, compared to controls, patients with overt diabetes had comparable ST2 levels (p = 0.179) and higher Gal-3 levels (p = 0.0002).

In a subset of patients (N = 26), we compared the levels of sST2 and Gal-3 in plasma *vs.* serum and we found higher levels in serum (p = 0.002 for both molecules; median values: sST2, 12.65 vs. 13.88 ng/mL; Gal-3 2.98 vs. 3.49 ng/mL], with a good correlation between values in the two sample types (sST2, rho = 0.912, p < 0.001; Gal-3, rho = 0.713, p < 0.001) (Additional file 1: Fig. S1).

Patients randomized to liraglutide or lifestyle counselling were comparable for most of the clinical and biochemical characteristics. The only significant between-arm differences despite randomization were triglyceride levels (p = 0.026), waist circumference (p = 0.040), VAT (p = 0.046) and sST2 (p = 0.008), all higher in the liraglutide arm (Table 1).

### Baseline sST2 and Gal-3 levels in relation to baseline metabolic variables

Baseline sST2 plasma levels were correlated directly with fasting insulin (rho = 0.391, p = 0.014) and VAT (rho = 0.376, p = 0.018) (Additional file 1: Table S1).

Baseline Gal-3 plasma levels correlated inversely with waist to hip ratio (WHR) (rho = − 0.455, p = 0.004) and directly with IL-6 (rho = 0.40, p = 0.023) (Additional file 1: Table S1).

### Baseline sST2 and Gal-3 levels in relation to baseline hs-TnI as a marker of myocardial involvement

**Fig. 3:**
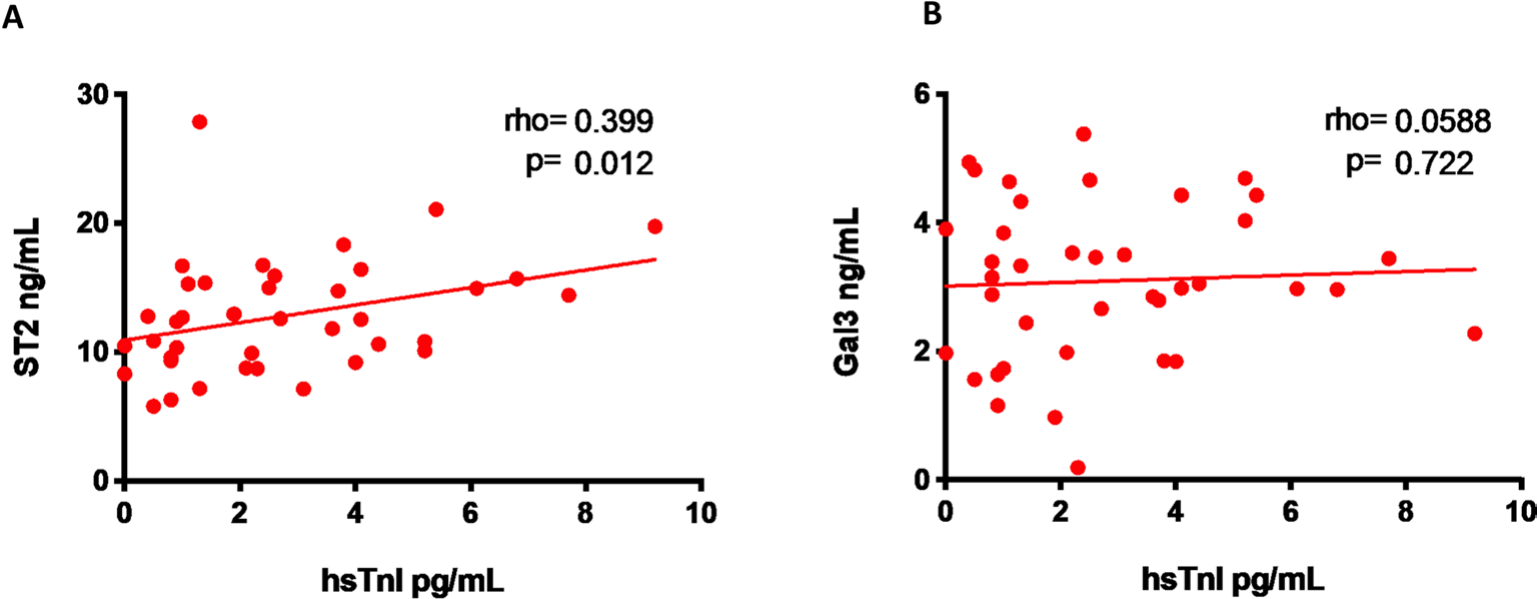
Baseline correlation between serum hs-TnI and plasma sST2 (**A**) and Gal-3 (**B**)

Baseline serum hs-TnI levels were correlated directly with sST2 (rho = 0.399, p = 0.012) (Fig. 3A), but not with Gal-3 (rho = 0.058 p = 0.722) plasma levels (Fig. 3B).

### Effects of intervention

**Fig. 4:**
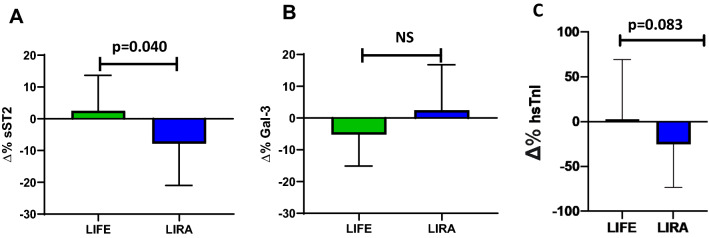
Effects of intervention on plasma sST2, Gal-3 levels and serum hs-TnI. ∆%changes of sST2 (**A**), Gal-3 (**B**) and serum hs-TnI (**C**) in the liraglutide or lifestyle arm after achievement of the weight loss target. P-value: between-group difference in ∆%sST2, ∆%Gal-3 and ∆%hs-TnI values adjusted for baseline waist circumference, baseline triglycerides and baseline VAT

At the end of the intervention period (i.e. after achievement of the weight loss target), we observed a significant reduction in sST2 levels in the liraglutide arm (− 8.99%, 95%CI − 15.8% to − 0.1%; p = 0.048) but not in the lifestyle arm (4.42%, 95%CI − 6.6% to 13.2%; p = 0.49) with a significant between-group difference in ∆%sST2 adjusted for basal triglycerides, basal VAT and basal waist circumference (beta = − 14.95, standard deviation 6.86, p = 0.037) (Fig. 4A). In contrast, Gal-3 levels were not affected by intervention in any of the two arms (Fig. 4B).

Moreover, overall (n = 40), a 3% (95%CI − 29% to 23%, p  = 0.81) reduction in serum hs-TnI was observed after intervention, due to a 19% (95%CI − 27% to 66%; p = 0.29) increase in the lifestyle arm, and a significant 25% decrease (95%CI − 48% to − 2%; p = 0.033) in the liraglutide arm, with a between-group difference in ∆%hs-TnI at the limits of significance, both unadjusted (p = 0.080) and adjusted for age, sex, basal triglycerides, basal VAT and basal waist circumference (beta = − 0.53, standard deviation 0.30, p = 0.083) (Fig. 4C)*.* We also found a direct correlation between baseline sST2 (rho = 0.399, p = 0.012) but not Gal-3 (rho = 0.058 p = 0.722) plasma levels and changes in serum high-sensitivity troponin I (data not shown). Thus, the higher ST2 at baseline, the lower the reduction in serum hs-TnI after intervention.

### Predictive role of Gal-3 and sST2 on metabolic variables

**Fig. 5:**
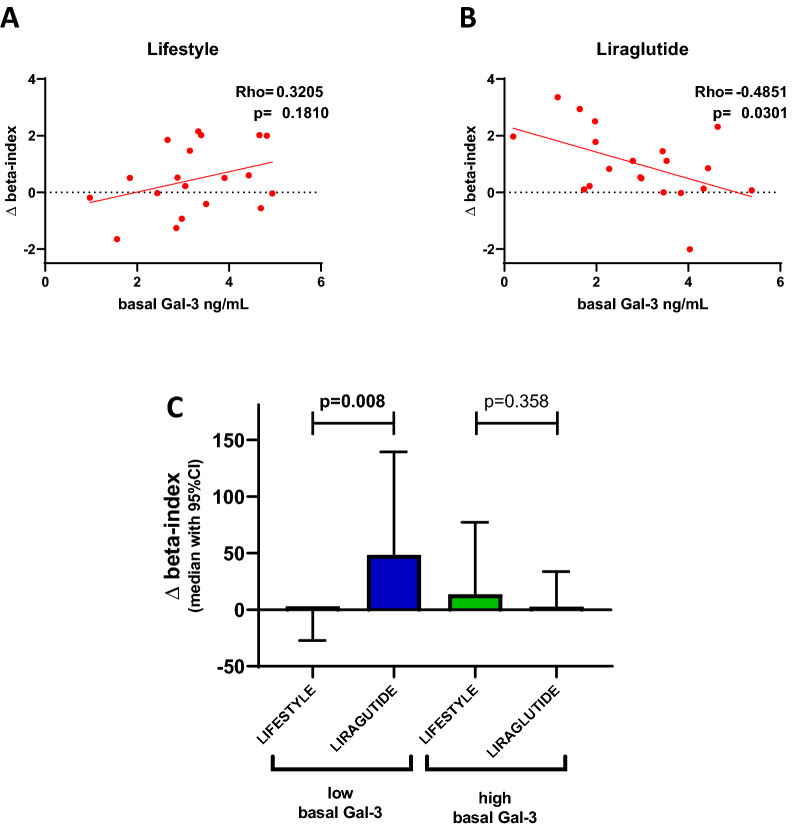
Predictive role of Gal-3 levels. Correlation between baseline levels of Gal-3 with ∆beta-index in the lifestyle (**A**) and in the liraglutide arm (**B**). **C** ∆beta-index in the liraglutide and in the lifestyle arm in patients with baseline Gal-3 levels above or under the median

In the liraglutide arm, but not in the lifestyle arm, we found that baseline levels of Gal-3 correlated inversely with the ∆beta-index (rho = − 0.485, p = 0.030; rho = 0.320, p = 0.181 respectively) (Fig. 5A, B).

Change in beta-index was similar in patients randomized to liraglutide compared with lifestyle (p = 0.11). However, by dividing patients in two groups according to baseline Gal-3 levels (above or under the median) we found that in patients with lower-than median Gal-3 levels there was a significant elevation of beta-index in those randomized to liraglutide compared with lifestyle, whereas such effect was not manifest in patients with higher-than median Gal-3 levels (p for difference of effect = 0.008) (Fig. 5C).

As for sST2, we also found a negative correlation between Gal 3 and delta beta-index (rho = − 0.481, p = 0.032). However, in the case of ST2, change in beta-index in the two arms was independent of ST2 levels above or below the median (p for difference of effect = 0.093).

### OGTT with frequent sampling

**Fig. 6:**
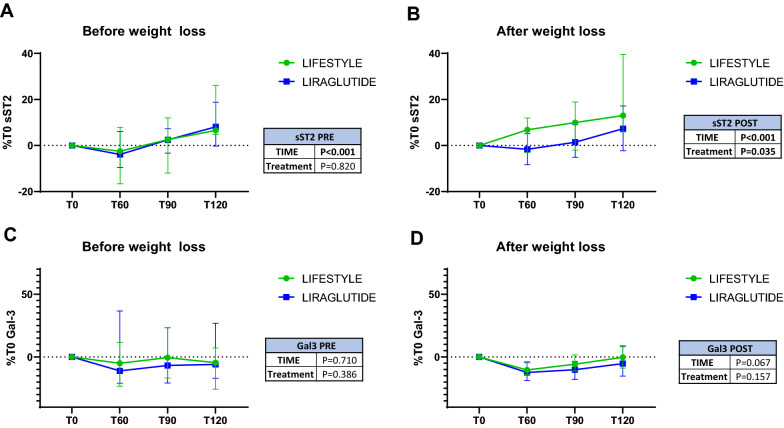
Effect of intervention on plasma sT2 and Gal-3 levels during OGTT. Levels of sST2 and Gal-3 during OGTT in lifestyle and liraglutide arm before (**A**, **C**) and after (**B**, **D**) weight loss

Before intervention, a 75 g oral glucose load led to an increase in sST2 levels over a period of 120 min (11.1%, p < 0.001). As expected, patients randomized to the two arms of treatment experienced a comparable increase in sST2 levels (12.2% lifestyle, 10.3% liraglutide p = 0.82) (Fig. 6A).

After intervention we still observed an increase of sST2 after the glucose load over time (14.1%, p < 0.001) in both arms, but this effect was less pronounced in patients randomized to liraglutide (21.4% lifestyle, 8.4% liraglutide, between-group p = 0.035) (Fig. 6B). Either before or after weight loss, the glucose load did not induce any change in Gal-3 levels in either arm (Fig. 6C, D).

## Discussion

In this sub-study of a previous randomized trial we evaluated the effect of liraglutide per se, regardless of the concurrent weight loss effect, on circulating sST2 and Gal-3, two validated markers of cardiac fibrosis and inflammation, as suggested by in vitro and in vivo experimental evidence [15, 16]. The main findings of this work were that (I) liraglutide treatment was associated with a significant 9% reduction in sST2 levels, and notably, no effect was observed in subjects achieving the same weight loss through lifestyle intervention; (II) an attenuated sST2 response during OGTT was also observed at the end of liraglutide-, but not lifestyle-induce weight loss; (III) Gal-3 levels were elevated in T2DM as compared with controls and may be used as a prognostic marker to predict the improvement of beta cell function induced by liraglutide and thus to identify patients which may benefit most from such therapy.

We analytically evaluated the two biomarkers and observed a good correlation between plasma and serum levels for both sST2 and Gal-3. However, the absolute levels were higher in serum *vs.* plasma and the major difference was observed for Gal-3. The additional Gal-3 in serum may be released by platelets which do express this molecule [19], and ideally, platelet-poor plasma should be used when analyzing this marker.

Previously, Yilmaz et al. reported that Gal-3 could be an independent predictor of diabetes [20] and Li et al. reported elevated Gal-3 levels in obese versus lean individuals, positively correlated with insulin resistance as assessed by HOMA score [17]. However, to this end, data on Gal-3 in diabetes are scarce or lacking. Herein we showed higher Gal-3 levels in patients with prediabetes and T2DM compared to control. In an experimental study in gene-modified mice it was shown that Gal-3 can bind directly to the insulin receptor and inhibit downstream insulin receptor signaling, contributing to decreased insulin signaling and insulin resistance and, at the same time, promote adipose tissue inflammation [21]. In addition, Gal-3 can interact with lipopolysaccharides (LPS) in settings characterized by low-grade endotoxemia, as reported in patients with prediabetes [21, 22], and trigger neutrophil activation [23] and possibly platelet activation.

On the contrary, we did not find any differences in sST2 levels between controls and patients with diabetes. Older age of our controls *vs.* patients can hardly explain this finding, since sST2 has been shown to be poorly influenced by age, and to predict outcome regardless of age [24].

In a previous study sST2 was increased in patients with diabetes *vs.* normal subjects [25], but our patients were in an early stage of the disease (< 12 months since diagnosis) and differences in sST2 levels may appear later. Indeed, sST2 is mainly elevated in patients with symptomatic CVD, reflecting myocardial wall stress and activation of fibrotic and inflammatory pathways [26, 27]. However, high levels are associated with poor prognosis also in the general population [28] and a reduction in sST2 may be beneficial despite levels being in the normal range.

Interestingly, we found an association between sST2 levels and metabolic features such as fasting insulin and VAT. These data are in line with the hypothesis that sST2 may be one link between obesity and diabetes development [29]. Adipose tissue has been identified as a source of sST2 and the induction of sST2 in a mouse model led to higher insulin secretion and exacerbated adipose tissue inflammation and insulin resistance [29].

In addition, we found that circulating ST2 significantly increased after 2-h glucose tolerance test, an increase mitigated at the end of the intervention period after liraglutide- but not lifestyle changes-induced weight loss. Thus, although our study showed no elevation of sST2 levels in T2DM, our findings relate this molecule to metabolic disturbances of relevance for diabetes.

Obesity is associated with cardiac dysfunction and activation of pro-fibrotic signaling pathways may lead to cardiac fibrosis. It has been demonstrated that these mechanisms may be reversible [30]. However, in the current study we showed that whereas 7% of weight loss achieved by lifestyle did not affect the levels of the profibrotic marker sST2, the same degree of weight loss in patients treated with liraglutide reduced sST2 levels, suggesting that this is a specific effect of this drug, at least in our patient population.

In contrast to the modest weight loss achieved in our study in either arm, a drastic body weight reduction associated with bariatric surgery led to a significant reduction in sST2 especially in diabetic patients [31]. This is probably related to the profound cardiometabolic reprogramming induced by bariatric surgery [32].

Interestingly, a large, randomized study reported no effect of canagliflozin, an antihyperglycemic drug belonging to a different class (sGLT2 inhibitors), on either sST2 or Gal-3, despite improvement in glycemic control [33]. Thus, liraglutide may act by modulating different pathophysiological mechanisms, independently, at least in part, of anti-hyperglycemic effects, potentially also exerting beneficial effects on myocardial fibrosis/injury [34].

In this regard, we found a direct correlation between serum hs-TnI levels and sST2; and a significant reduction in serum hs-TnI in the liraglutide arm. Troponins are specific biomarkers of myocardial injury. However, they also rise in other acute and chronic situations and even in apparently healthy populations, and high cardiac troponin concentrations within the normal range are independent predictors of vascular events and death [35–38]. sST2 expression occurs mostly in endothelial cells in response to tissue damage and inflammation [7], and exerts deleterious effects on the heart, where it abolishes the cardioprotective effects of the IL-33/ST2 interaction [39]. Thus, it is not surprising that sST2 and hs-TnI are inversely correlated and ST2 reduction translates into reduced hs-TnI.

While the molecular mechanisms underlying the effects of liraglutide on sST2 remain uncharacterized, we can speculate that it may involve pathways related to inflammation, oxidative stress and/or endothelial dysfunction [40]. GLP-1 receptor is expressed in cardiomyocytes, although to date it is uncertain what its exact function in humans is [41].

At variance, experimental evidence suggests that endothelial GLP-1 receptor may mediate cardiovascular protection by liraglutide in mice with arterial hypertension [42]. In vivo data in the setting of obesity clearly show that GLP-1RA signaling inhibits allergen-induced IL-33 release in the airway [43]. Thus, the IL-33/ST2 signaling may be favorably affected by this class of drugs, but further studies are needed to verify this hypothesis.

Gal-3 levels were not affected by weight loss achieved by lifestyle change or liraglutide treatment. However, low basal Gal-3 levels were predictive for the efficacy of liraglutide to improve beta-cell function. A possible role of Gal-3 as a predictor of successful therapy in patients with T2DM has been previously discussed [44]. Since liraglutide seems most effective in patients with the highest remaining β‐cell function [45, 46] our hypothesis is that low basal Gal-3 levels may serve as biomarkers to identify this subset of patients. This is supported by a recent experimental model of obesity-induced diabetogenesis, showing that Gal-3 overexpression facilitates β-cell damage, enhances oxidative stress and beta-cell apoptosis [47].

Limitations of this study include lack of imaging cardiac function markers, such as MRI and echocardiography and soluble markers of cardiac function (i.e., natriuretic peptides) as well as clinical cardiovascular endpoints. The number of patients were also relatively low which may weaken our conclusions. We also lack data on IL-33 which is related to sST2 function. Strengths include the randomized designed of the study, excluding the confounding effect of weight loss on the markers in study, and the state-of-the-art method to detect beta-cell function in vivo. Moreover, the effect of the acute increase of blood glucose after OGTT allows making assumptions about the role of acute hyperglycemia on the circulating levels of both molecules in study.

## Conclusions

This randomized study on obese patients with prediabetes or early T2DM, suggests that the reduced CV risk observed with liraglutide treatment may be at least partially due to the prevention/improvement of cardiac fibrosis as revealed by the reduction of sST2 levels. Although further clinical studies are necessary to fully elucidate the underlying mechanisms, this finding adds a piece of the puzzle justifying the cardiovascular benefit of liraglutide. Importantly, however, such studies should also include directly measurements of cardiac function and the degree of fibrosis. Moreover, Gal-3 levels may be used as a prognostic marker to identify the patient that will benefit most from liraglutide therapy, in terms of improvement in beta cell function. Thus, circulating galectin-3 may be feasible as a biomarker-guided therapy in patients with T2DM.

## Supplementary Information

**Additional file 1.** 1. Methods 1.1 Eligibility criteria 2. Supplemental Table 1 3. Supplemental Figure 1.

## Data Availability

The datasets analysed during the current study are available from the corresponding author on reasonable request.

## Abbreviations

sST2: Soluble suppression of tumorigenesis-2
Gal-3: Galectin-3
T2DM: Type 2 diabetes mellitus
IFG: Impaired fasting glucose
IGT: Impaired glucose tolerance
ELISA: Enzyme-linked immunosorbent assays
IL-1: Interleukin-1
GLP-1: Glucagon-like peptide-1
VAT: Visceral adipose tissue
SAT: Subcutaneous adipose tissue
OGTT: Oral glucose tolerance test
BMI: Body mass index
