# Supplementary material for: Effects of liraglutide vs. lifestyle changes on soluble suppression of tumorigenesis-2 (sST2) and galectin-3 in obese subjects with prediabetes or type 2 diabetes after comparable weight loss
Source: Cardiovasc Diabetol. 2022 Mar 11;21:36. doi: 10.1186/s12933-022-01469-w (PMC8917620; doi:10.1186/s12933-022-01469-w)
Supplement: Supplementary file 1 — Additional file 1. 1. Methods 1.1 Eligibility criteria 2. Supplemental Table 1 3. Supplemental Figure 1. [file 12933_2022_1469_MOESM1_ESM.docx]

Effects of liraglutide vs. lifestyle changes on sST2 and Galectin-3 in obese subjects with prediabetes or type 2 diabetes after comparable weight loss.

Paola Simeone^1^*, Romina Tripaldi^1^*, Annika Michelsen^2*^, Thor Ueland^2^, Rossella Liani^1^, Sonia Ciotti^1^, Kåre I. Birkeland^3^, Hanne L Gulseth^4^, Augusto Di Castelnuovo^5^, Francesco Cipollone^1^, Pål Aukrust^2^, Agostino Consoli^1^, Bente Halvorsen^2^**, Francesca Santilli^1^**

*Joint first authors

**Joint last authors

^1^Department of Medicine and Aging, and Center for Advanced Studies and Technology (CAST), Via Luigi Polacchi, Chieti, Italy

^2^ Research Institute of Internal Medicine, Oslo University Hospital Rikshospitalet, University of Oslo, Oslo, Norway

^3^ Department of Transplantation Medicine, Institute of Clinical Medicine, University of Oslo and Oslo University Hospital, Norway

^4^ Department of Endocrinology, Morbid Obesity and Preventive Medicine, Oslo University Hospital, Oslo, Norway, Department of Chronic Diseases and Ageing, Norwegian Institute of Public Health, Oslo, Norway

^5^ Mediterranea Cardiocentro, Napoli, Italy

**Corresponding author contact information**

Francesca Santilli at the Center for Advanced Studies and Technology (CAST), “G. D’Annunzio” University Foundation, Via Luigi Polacchi, 66013 Chieti, Italy. Tel: +39-0871-541312. Fax: +39-0871-541261. E-mail: [francesca.santilli@unich.it](mailto:francesca.santilli@unich.it)

**Additional file**

**Methods**

*Eligibility criteria*

We enrolled subjects with BMI >30, with a diagnosis of IGT or IFG or type 2 diabetes since less than 12 months, according to the American Diabetes Association (ADA) Guidelines [1]. At the time of enrolment, all patients were treated by diet therapy plus metformin at the highest tolerated dose (up to 3000 mg/day).

*Exclusion criteria*

Exclusion criteria included type 1 DM, BMI<30 kg/m^2^, DM diagnosed since >12 months, treatment with oral antidiabetic agents (except metformin) or insulin within the last three months, uncontrolled hypertension (systolic/diastolic blood pressure >160/90 mmHg), significant co-morbid diseases such as kidney disease with glomerular filtration rate below 60 ml or liver disease (AST or ALT twice above the upper normal range), pregnancy or lactation; female of child-bearing potential intending to become pregnant or is not using adequate contraceptive methods while sexually active; any contraindication to liraglutide (known or suspected hypersensitivity to liraglutide or related products, previous acute pancreatitis or chronic pancreatitis, inflammatory bowel disease, gastrointestinal surgery (e.g. gastric bypass), heart failure Class NYHA III-IV); personal history or family history of medullary thyroid carcinoma or personal history of multiple endocrine neoplasia type 2; claustrophobia; Metal implants or other contraindications for MRI; recent participation in other research projects within the last 3 months or participation in 2 or more projects in one year.

Patients not achieving the weight loss goal within 15 months since the initiation of the randomized treatment, as well as those not completing the study for decision of the patient and/or of the Investigator, were considered withdrawn from the study, and were replaced, in order to attain the anticipated sample size.

1. Classification and diagnosis of diabetes. Diabetes Care. 2015;38:S8–16.

**Supplemental Table 1. Baseline correlation between sST2 and Gal-3 and metabolic markers**

|  | **Insulin** | **VAT** | **WHR** | **IL-6** |
| --- | --- | --- | --- | --- |
| **ST2** | Rho=0.391  **P=0.014** | Rho=0.376  **P=0.018** | Rho=-0.062  P=0.709 | Rho=0.100  P=0.588 |
| **Gal 3** | Rho=-0.049  P=0.767 | Rho=-0.118  P=0.476 | Rho=-0.455  **P=0.004** | Rho=0.402  **P=0.023** |

*Abbreviations*:, WHR= waist-hip ratio, VAT= visceral-adipose-tissue, IL-6 interleukin-6.


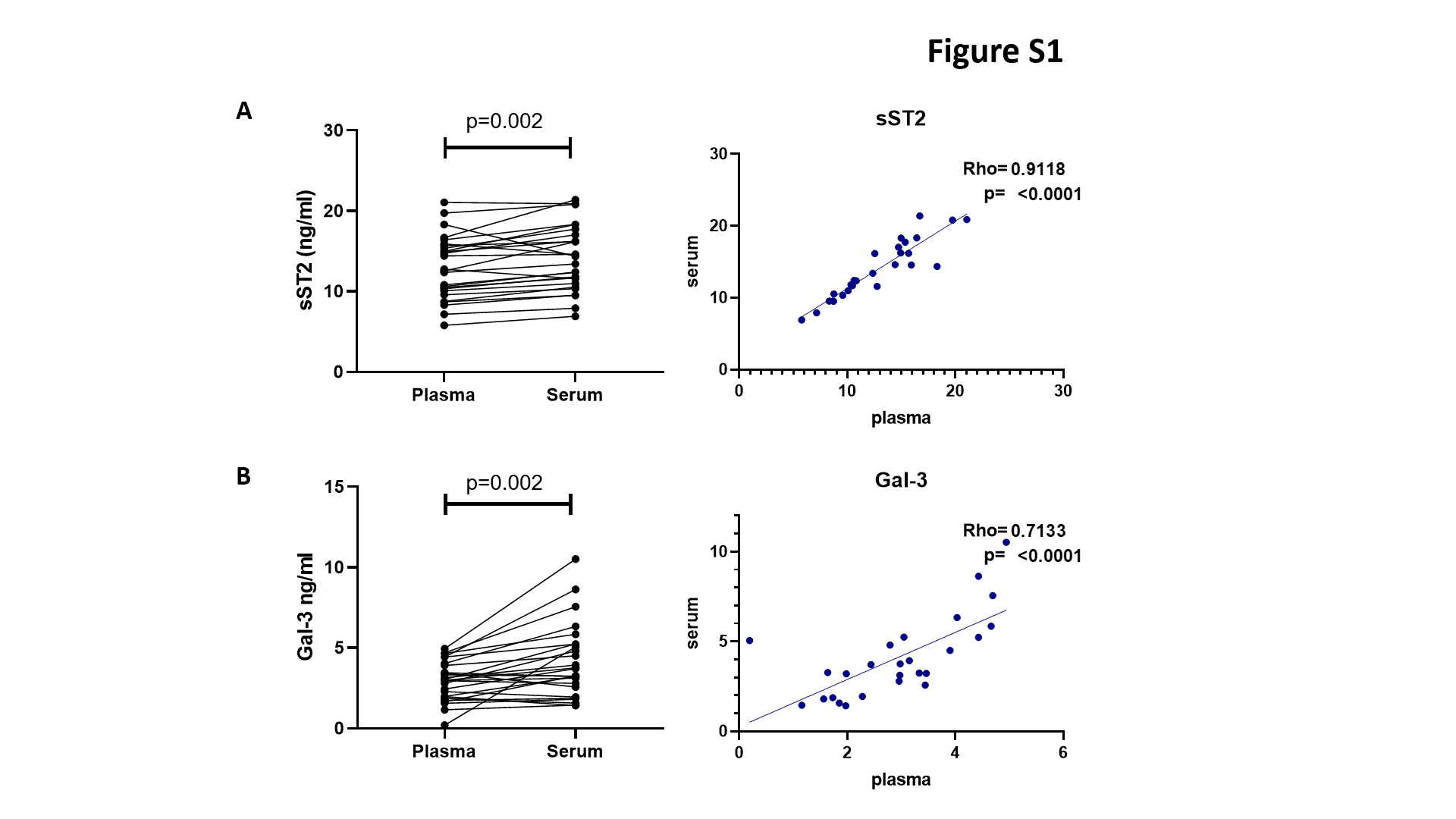


**Figure S1. Baseline sST2 and Gal-3 levels.** Comparison between levels of sST2 (A) and (B) Gal-3 in plasma *vs* serum in the cohort of patients.
